# Comparative Effects of Two Hyaluronic Acid-Based Formulations on Cellular Repair Mechanisms in Human Keratinocytes

**DOI:** 10.3390/ijms262412090

**Published:** 2025-12-16

**Authors:** Robert Chmielewski, Agata Lebiedowska

**Affiliations:** 1 Prime Clinic, Topiel 12, 00-342 Warsaw, Poland; cmvitamed@gmail.com; 2 Positive Pro-Aging Foundation, Topiel 12, 00-342 Warsaw, Poland; 3 URGO Aesthetics Department, URGO Sp. z o.o., Aleje Jerozolimskie 142 B, 02-305 Warsaw, Poland; 4 Department of Basic Biomedical Science, Faculty of Pharmaceutical Sciences in Sosnowiec, Medical University of Silesia, 40-055 Katowice, Poland

**Keywords:** hyaluronic acid, trehalose, molecular weight, keratinocytes, cellular repair, re-epithelialization, scratch assay, aesthetic medicine

## Abstract

Hyaluronic acid formulations are widely used in aesthetic and regenerative medicine, yet molecular weight-dependent effects on cellular repair remain incompletely characterized. This study investigated two hyaluronic acid formulations—low- and medium-high-molecular-weight ranges with trehalose (LMHMW-HA; 200–400 and 1200–1500 kDa) and high-molecular-weight range (HMW-HA; 1800–2600 kDa)—on keratinocyte repair using an in vitro scratch assay. Human keratinocyte monolayers were treated with various concentrations, and repair dynamics were monitored over 48 h through microscopy and quantitative inter-edge distance analysis. Both formulations significantly enhanced gap closure compared to controls without cytotoxic effects. LMHMW-HA promoted gradual closure over 48 h with increased cellular density, indicating sustained proliferation and migration. HMW-HA induced faster closure at approximately 30 h, accompanied by transient pericellular swelling consistent with hydration-mediated edge approximation. These findings indicate that LMHMW-HA and HMW-HA promote repair through distinct patterns: LMHMW-HA was associated with gradual closure and increased cellular density consistent with proliferation-driven repair, while HMW-HA induced rapid closure with transient pericellular swelling consistent with hydration-mediated effects. These preliminary observations suggest complementary repair mechanisms and provide a foundation for future mechanistic investigations. The molecular weight-tailored approach combining HA with trehalose offers therapeutic potential for aesthetic and regenerative medicine applications requiring enhanced tissue repair.

## 1. Introduction

Cellular repair mechanisms are fundamental biological processes that restore tissue integrity following injury through coordinated migration, proliferation, and matrix remodeling. In epithelial tissues, keratinocytes play a central role in re-establishing barrier function by migrating from wound edges to cover denuded areas—a process termed re-epithelialization [1,2,3,4,5,6]. This cellular response involves complex signaling cascades regulated by growth factors, cytokines, and interactions between cells and the extracellular matrix (ECM) [1,7,8,9]. Fibroblasts contribute to repair by synthesizing and remodeling ECM constituents, including collagens and proteoglycans, concurrently influencing keratinocyte growth and maturation via release of bioactive molecules including interleukin-1 (IL-1), keratinocyte growth factor (KGF), and granulocyte-macrophage colony-stimulating factor (GM-CSF) [10,11]. The equilibrium among these reparative mechanisms dictates the trajectory of healing, either toward normal tissue architecture or fibrotic pathology. Understanding the molecular pathways regulating keratinocyte migration and proliferation is thus critical for designing interventions that promote tissue repair in aesthetic and reconstructive medicine.

**Figure 1:**
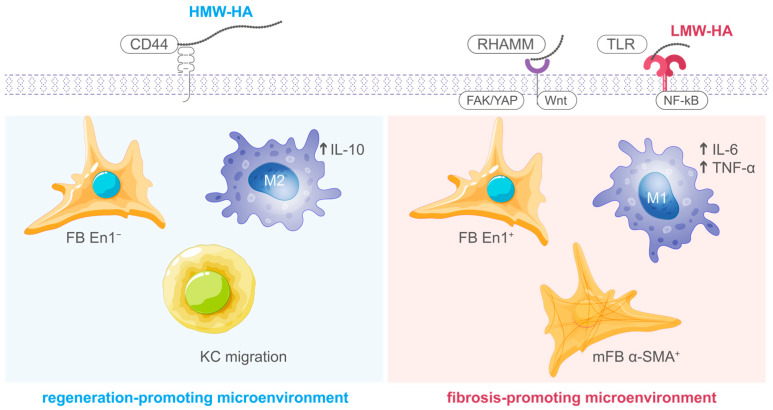
Molecular weight-dependent mechanisms of hyaluronic acid in cellular repair. HMW-HA (left) and LMW-HA (right) engage distinct receptors and polarize macrophages toward regenerative (M2, IL-10) or inflammatory (M1, IL-6/TNF-α) phenotypes, respectively. FB En1 = engrailed-1 positive fibroblasts; KC = keratinocytes; mFB α-SMA+ = myofibroblasts. The molecular weight-dependent effects of hyaluronic acid on cell migration and motility have been demonstrated across various cell types and tissue contexts. Low-molecular-weight HA has been shown to promote endothelial cell spreading and migration through enhanced focal adhesion assembly [16], while LMW-HA has also been reported to facilitate cell transmigration across lymphatic endothelium by disrupting intercellular barrier function [17]. These findings across different cell types suggest that the pro-migratory effects of LMW-HA may represent a conserved biological response. In vitro scratch wound assays have demonstrated molecular weight-dependent effects of hyaluronic acid on keratinocyte repair mechanisms. Kawano et al. showed that high-molecular-weight HA (2290 kDa) at 0.1% concentration significantly promoted keratinocyte wound closure compared to medium- (987 kDa) and low-molecular-weight (8 kDa) fractions, with migration-promoting effects observable within 6 h and enhanced gap closure at 24 and 48 h [6]. Similarly, low-molecular-weight HA (~18 kDa) has been shown to significantly enhance keratinocyte migration in scratch assays through activation of HIF-1α/VEGF signaling pathways [18]. However, comparative studies evaluating commercially available formulations combining different molecular weight ranges with bioprotective agents such as trehalose remain limited, particularly regarding their distinct kinetic profiles and morphological patterns in cellular repair models. ↑ indicates upregulation. Blue and red shading distinguish regeneration-promoting (HMW-HA) and fibrosis-promoting (LMW-HA) microenvironments, respectively.

Hyaluronic acid (HA) represents a non-sulfated glycosaminoglycan consisting of repetitive disaccharide moieties containing D-glucuronic acid and N-acetyl-D-glucosamine [12]. This ubiquitous extracellular matrix constituent exerts essential regulatory functions during cellular repair. HA exhibits molecular weight-dependent bioactivity, whereby high-molecular-weight (HMW-HA; >1000 kDa) and low-molecular-weight (LMW-HA; <500 kDa) variants demonstrate divergent functional characteristics [13]. HMW-HA, which predominates in physiologically normal tissues, exerts anti-inflammatory and cytoprotective actions by preserving ECM water content, supporting cell–matrix interactions through CD44 receptor binding, and inhibiting excessive inflammatory responses [9,14]. In contrast, LMW-HA fragments generated during tissue injury or enzymatic degradation function as endogenous danger signals that activate innate immune receptors, particularly Toll-like receptors 2 and 4 (TLR2/4), thereby initiating pro-inflammatory and pro-angiogenic responses essential for the early phases of tissue repair [13,15]. This molecular weight-dependent duality allows HA to function as a dynamic regulator that modulates the transition from injury-induced inflammation to matrix remodeling and tissue stabilization (Figure 1) [9,12].

Trehalose, a non-reducing disaccharide formed by two glucose units linked by an α,α-1,1-glycosidic bond, is recognized as a potent bioprotective agent exhibiting diverse functions in cellular stress response and tissue repair [19]. Beyond its well-established function as a chemical chaperone that stabilizes proteins and membranes under oxidative stress conditions, trehalose actively modulates cellular repair mechanisms through several distinct pathways [20,21,22]. Trehalose promotes autophagy—a critical cellular quality control process that removes damaged organelles and protein aggregates—thereby supporting the metabolic adaptation required for sustained repair activity [23,24]. Furthermore, trehalose exerts protective effects against advanced glycation end-products (AGEs), which accumulate during oxidative stress and aging, disrupting normal ECM architecture and impairing cellular migration [19]. Recent evidence indicates that trehalose is capable of inducing fibroblasts to establish a prohealing senescence-like phenotype defined by cell cycle arrest and enhanced growth factor secretion, thereby promoting tissue regeneration by creating a favorable paracrine environment for keratinocyte proliferation and migration [25]. When combined with hyaluronic acid, trehalose demonstrates synergistic effects that enhance both the stability of HA within the ECM and its bioactivity, creating an optimal microenvironment for keratinocyte migration and proliferation while simultaneously mitigating oxidative damage and glycation-induced tissue dysfunction [19,26,27].

Although the distinct biological functions of HMW-HA and LMW-HA in tissue repair are established, the relative impacts of formulations combining different molecular weight ranges of HA with trehalose on keratinocyte repair mechanisms are not yet fully defined. Therefore, the present study aimed to provide preliminary characterization comparing the effects of two distinct hyaluronic acid-based formulations—one containing low- and medium-high-molecular-weight range HA combined with trehalose, and another containing high-molecular-weight HA—on cellular repair processes in human keratinocytes using an in vitro scratch wound model. While these formulations differ in both molecular weight composition and the presence of trehalose, the findings may provide insights into their distinct mechanisms of action and potential applications in aesthetic and regenerative medicine.

## 2. Results

### 2.1. Low- and Medium-High-Molecular-Weight Range HA Formulation (Resteem X)

**Figure 2:**
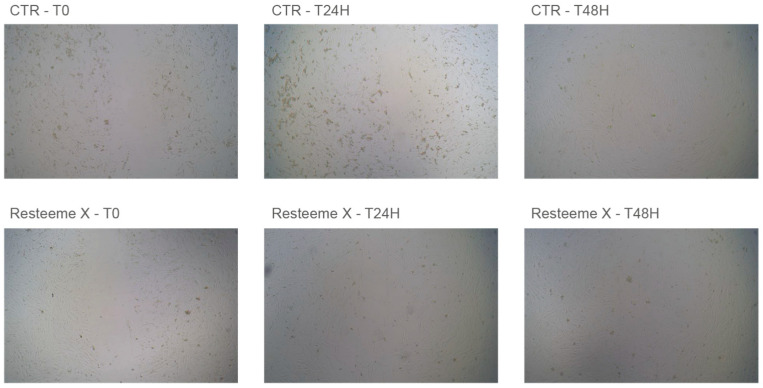
Representative micrographs of keratinocyte cultures without product (CTR−; upper row) and after treatment with Resteem X (low- and medium-high-molecular-weight range HA + trehalose; lower row) at baseline (T0), 24 h (T 24 h), and 48 h (T 48 h). The acellular gap extends vertically through the center of each image. Micrographs were captured at 4× magnification.

Application of the low- and medium-high-molecular-weight range hyaluronic acid formulation containing trehalose (Resteem X; hereafter referred to as the LMHMW-HA formulation with trehalose) promoted visible keratinocyte migration and proliferation in the cellular repair model. All measurements represent mean values ± standard deviation (SD) derived from three separate experiments (*n* = 3). Representative micrographs (Figure 2) illustrate control (CTR−) and treated cultures at baseline (T0), 24 h, and 48 h. Progressive closure of the acellular gap was observed from lesion borders toward the center, and at 48 h the treated monolayers exhibited complete closure with a visibly higher cell density compared with controls.

At baseline (T0), the inter-edge distance averaged 691.4 ± 66.7 µm in controls.

After 2 h, distances were 685.9 ± 56.2 µm (−0.8%) in controls and 646.8 ± 81.5 µm (−2.5%), 649.4 ± 84.5 µm (−1.5%), and 665.6 ± 56.1 µm (−1.0%) for 0.5%, 0.25%, and 0.1% Resteem X, respectively.

After 8 h, controls measured 643.4 ± 31.4 µm (−6.9%), while the corresponding values for treated cultures were 512.9 ± 80.2 µm (−22.7%), 527.3 ± 33.9 µm (−20.0%), and 605.0 ± 31.3 µm (−10.0%) for 0.5%, 0.25%, and 0.1%.

**Figure 3:**
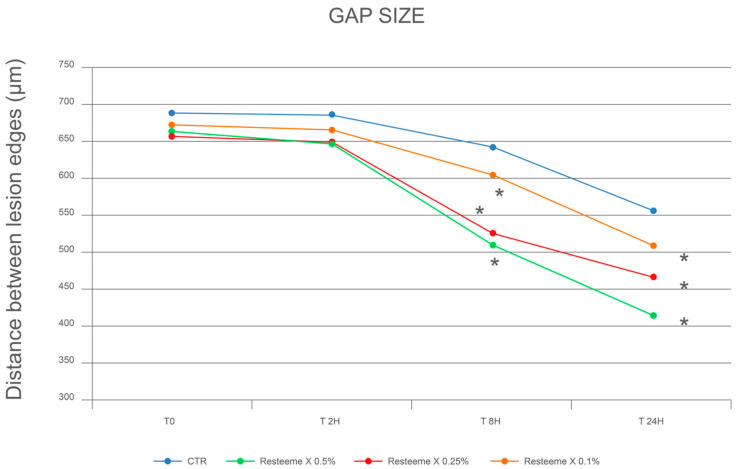
Quantitative analysis of inter-edge distance reduction in keratinocyte monolayers treated with Resteem X compared to untreated controls (CTR−) after 2, 8, and 24 h (mean ± SD). (*) indicate statistically significant differences compared to untreated control (*p* < 0.05). Standard deviations for all data points are provided in Table 1 and Table 2.

After 24 h, controls were 556.3 ± 37.2 µm (−19.5%), compared with 416.4 ± 69.3 µm (−37.2%), 467.4 ± 83.2 µm (−29.1%), and 510.7 ± 47.2 µm (−24.1%) for the three concentrations (Figure 3; Table 1 and Table 2).

No cytotoxic alterations (e.g., vacuolization or detachment) were observed at any concentration. Complete gap closure was observed at approximately 48 h for all tested concentrations of Resteem X. Visual inspection confirmed full gap closure at this time point, although photographic documentation was limited to baseline, 2 h, 8 h, 24 h, and 48 h intervals. At the highest concentration, a slight tightening of the monolayer edges was visible, consistent with a more condensed alignment and enhanced cellular density.

### 2.2. High-Molecular-Weight Range HA Formulation (Resteem L)

Application of the high-molecular-weight range hyaluronic acid formulation (Resteem L; hereafter referred to as the HMW-HA formulation) induced a faster and morphologically distinct repair response in keratinocyte monolayers.

**Figure 4:**
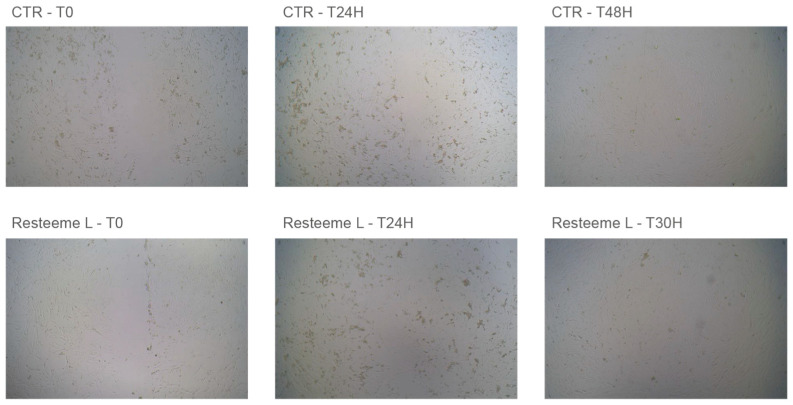
Representative micrographs of keratinocyte cultures without product (CTR−; upper row) and after treatment with Resteem L (high-molecular-weight range HA; lower row) at baseline (T0), 24 h (T24 h), and 30 h (T30 h). The acellular gap extends vertically through the center of each image. Micrographs were captured at 4× magnification.

Figure 4 presents representative micrographs of control (CTR−) and treated cultures at baseline (T0), 24 h, and 30 h, showing complete closure of the acellular gap in treated cultures.

During the first few hours, localized pericellular swelling was observed in treated wells, followed by compact, continuous monolayer formation and uniform cellular alignment.

At baseline (T0), the inter-edge distance averaged 691.4 ± 66.7 µm in controls.

After 2 h, distances were 685.9 ± 56.2 µm (−0.8%) in controls and 649.0 ± 38.9 µm (−0.9%), 653.9 ± 90.8 µm (−0.7%), and 661.7 ± 51.9 µm (−0.2%) for 0.25%, 0.1%, and 0.05% Resteem L, respectively.

After 8 h, controls measured 643.4 ± 31.4 µm (−6.9%), while treated cultures reached 515.9 ± 44.5 µm (−21.3%), 555.0 ± 49.6 µm (−15.8%), and 619.7 ± 77.6 µm (−6.5%), respectively.

**Figure 5:**
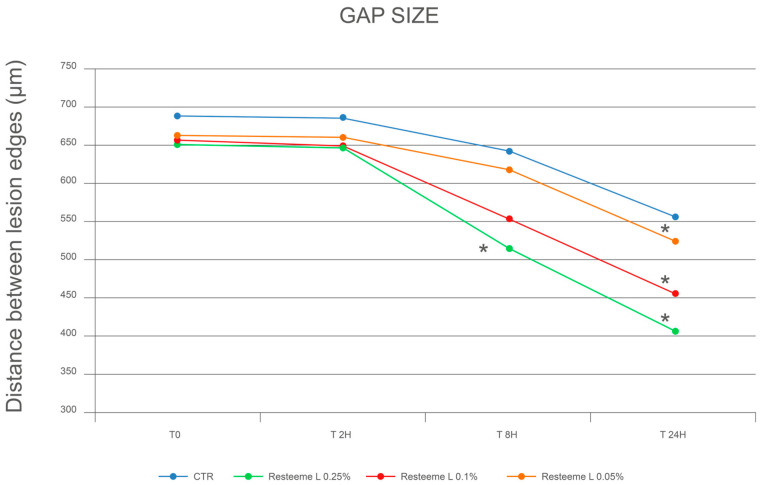
Quantitative analysis of inter-edge distance reduction in keratinocyte monolayers treated with Resteem L compared to untreated controls (CTR−) after 2, 8, and 24 h (mean ± SD). (*) indicate statistically significant differences compared to untreated control (*p* < 0.05). Standard deviations for all data points are provided in Table 3 and Table 4.

After 24 h, controls were 556.3 ± 37.2 µm (−19.5%), compared with 407.8 ± 40.0 µm (−37.8%), 456.1 ± 35.7 µm (−30.8%), and 525.0 ± 18.0 µm (−20.8%) for the three concentrations (Figure 5; Table 3 and Table 4).

No cytotoxic alterations or detachment were noted at any concentration. Complete gap closure was observed at approximately 30 h for all tested concentrations of Resteem L, earlier than observed for Resteem X. The exact time point between 24 h and 30 h was determined by visual inspection, as photographic documentation was performed at baseline, 2 h, 8 h, 24 h, and 30 h intervals. At higher concentrations, the pericellular edema observed initially was transient and corresponded with faster closure and increased cellular cohesion after 24 h.

## 3. Discussion

This study demonstrates two principal findings regarding hyaluronic acid formulations in keratinocyte repair. First, the LMHMW-HA formulation with trehalose (Resteem X, 200–400 and 1200–1500 kDa) significantly accelerated gap closure compared to untreated controls, achieving 37.2% reduction in inter-edge distance at 24 h (*p* < 0.001) and complete closure at 48 h with visibly increased cellular density. Second, the HMW-HA formulation (Resteem L, 1800–2600 kDa) induced even faster initial closure, reaching 37.8% reduction at 24 h (*p* < 0.001) and complete closure at approximately 30 h, accompanied by transient pericellular swelling. These findings address a critical knowledge gap in understanding how commercially available HA formulations with different molecular weight profiles and compositions promote cellular repair through distinct patterns—LMHMW-HA associated with gradual closure and increased cellular density consistent with proliferation-driven repair, while HMW-HA induced rapid closure with transient pericellular swelling consistent with hydration-mediated effects—with implications for indication-specific selection in aesthetic and regenerative medicine.

The LMHMW-HA formulation with trehalose (Resteem X) promoted gradual gap closure over 48 h with visibly increased cellular density in the final monolayer. These observations suggest sustained cellular proliferation and migration throughout the observation period. This pattern differs markedly from passive wound contraction and suggests active cellular engagement in the repair process. The intermediate closure time (~48 h) and increased cell density are consistent with LMHMW-HA’s capacity to stimulate both migratory and proliferative programs in keratinocytes, as previously demonstrated in studies showing enhanced expression of matrix metalloproteinases and growth factors following LMHMW-HA exposure [6,28]. Unlike formulations that primarily induce mechanical edge approximation, Resteem X appears to promote biologically active repair characterized by cellular recruitment and matrix remodeling, supporting its potential application in scenarios requiring enhanced tissue regeneration rather than rapid closure alone.

In contrast to the gradual repair pattern observed with LMHMW-HA, the HMW-HA formulation (Resteem L) induced faster gap closure with complete coverage achieved at approximately 30 h. The observed transient pericellular swelling during early time points is consistent with the high water retention capacity of HMW-HA, which creates localized hydration that facilitates mechanical approximation of lesion edges [12,14]. This rapid edge contraction represents a distinct mechanism from the proliferation-driven closure observed with LMHMW-HA, suggesting that HMW-HA primarily functions through matrix-mediated physical effects rather than direct cellular activation [9,14]. The faster closure time and absence of prolonged inflammatory-like activation are consistent with HMW-HA’s role in stabilizing repair processes and creating conditions favorable for physiological tissue remodeling [19,26].

The observed effects align with established molecular weight-dependent patterns in keratinocyte repair. Kawano et al. demonstrated that HMW-HA (2290 kDa) promoted keratinocyte wound closure with sustained effects observable from 6 to 48 h [6]. Our observation that HMW-HA (1800–2600 kDa) induced closure by approximately 30 h demonstrates a similar pattern of sustained closure, though the faster kinetics in our study may reflect differences in molecular weight range or experimental conditions. However, the transient pericellular swelling observed in our study suggests that closure mechanisms may vary with specific molecular weight ranges and formulation contexts. Similarly, Liu et al. showed that LMW-HA (~18 kDa) enhanced keratinocyte migration through HIF-1α/VEGF pathway activation [18], supporting the interpretation that the LMHMW-HA formulation (200–400 and 1200–1500 kDa) promotes biologically active repair involving cellular proliferation and migration. The inclusion of trehalose in the LMHMW-HA formulation may provide additional metabolic protection that sustains these repair processes under conditions of cellular stress.

The inclusion of trehalose in the LMHMW-HA formulation likely contributed to the observed repair-promoting effects by providing metabolic protection during the cellular stress associated with injury and repair. The combination of trehalose with low and medium-high molecular-weight range HA may enhance both the biological (migration, proliferation) components of keratinocyte repair while protecting cells from oxidative and glycation-related damage [19,25,26,27]. In contrast, the HMW-HA formulation without trehalose demonstrated effective repair promotion primarily through matrix-mediated physical effects, suggesting that high-molecular-weight HA alone is sufficient to induce rapid hydration-mediated closure. The complementary nature of these mechanisms—LMHMW-HA with trehalose providing biologically active repair and metabolic protection, versus HMW-HA providing structural stabilization—is consistent with recent findings demonstrating that different molecular weight HA formulations can address distinct phases of tissue repair [13,14,28].

An important consideration in interpreting these findings is that the two formulations were evaluated in separate experimental series using different concentration ranges (0.5–0.1% for Resteem X vs. 0.25–0.05% for Resteem L), reflecting empirically determined optimal non-cytotoxic doses for each molecular weight range. Consequently, direct quantitative comparison of efficacy between formulations was not the primary goal of this study; rather, the aim was to characterize the distinct mechanisms by which each formulation promotes cellular repair. Additionally, since Resteem X contains trehalose while Resteem L does not, the observed mechanistic differences may reflect both molecular weight effects and the bioprotective contribution of trehalose. A critical limitation of this study is that the mechanistic interpretation is based on morphological and kinetic observations from a scratch assay model, without direct molecular characterization of the underlying cellular pathways. While the observed patterns—increased cellular density with LMHMW-HA versus transient pericellular swelling with HMW-HA—are consistent with proliferation-driven and hydration-mediated mechanisms, respectively, definitive mechanistic conclusions would require additional experiments including proliferation assays (e.g., Ki-67 immunostaining, BrdU incorporation), migration-specific assays, analysis of matrix metalloproteinase expression and extracellular matrix remodeling, and evaluation of receptor engagement and signaling pathways. The present study therefore provides a preliminary characterization that establishes a foundation for future mechanistic investigations. The observed differences—gradual closure with increased cellular density for LMHMW-HA versus rapid edge contraction with transient pericellular swelling for HMW-HA—suggest complementary rather than competing mechanisms. This interpretation aligns with recent findings demonstrating that combined molecular weight HA formulations can address multiple phases of tissue repair sequentially: LMW-HA fragments initiate controlled inflammatory activation and cellular recruitment during early repair phases, while HMW-HA subsequently stabilizes the matrix and suppresses excessive inflammation during remodeling phases [13,14,28]. The complementary nature of these mechanisms has been previously demonstrated in ex vivo human skin models, where dual molecular weight HA formulations combined with trehalose exhibited superior anti-glycation protection and enhanced barrier function compared to single molecular weight preparations [26,27].

Several limitations must be acknowledged. First, this study employed a monolayer scratch assay, which models cellular repair mechanisms in a simplified two-dimensional system lacking the complexity of three-dimensional tissue architecture, dermal-epidermal interactions, immune cell participation, and vascularization present in physiological wound healing. Therefore, while the observed effects demonstrate cellular repair capacity in keratinocytes, extrapolation to in vivo wound healing must be made cautiously. Second, the absence of direct side-by-side comparison at identical concentrations limits conclusions regarding relative potency. Third, the study focused exclusively on keratinocyte responses without evaluating potential effects on fibroblasts, endothelial cells, or immune cells that contribute to tissue repair in vivo. Despite these limitations, the in vitro model provides valuable mechanistic insights into molecular weight-dependent effects of HA formulations on keratinocyte behavior, which may inform rational design of therapeutic approaches in aesthetic and regenerative medicine.

These findings may have practical implications for aesthetic and regenerative medicine. The LMHMW-HA formulation with trehalose, which promotes sustained cellular proliferation and migration, may be particularly suitable for: biorevitalization procedures aimed at improving dermal cellular density and extracellular matrix quality; post-procedural healing support following ablative or non-ablative laser treatments, where cellular recruitment and tissue regeneration are required; and management of atrophic scars or photoaged skin, where stimulation of fibroblast activity and matrix remodeling are therapeutic goals. In contrast, the HMW-HA formulation, which induces rapid hydration-mediated tissue stabilization, may be advantageous for: immediate structural support in dermal filling applications; rapid restoration of skin barrier function following superficial injury or inflammation; and maintenance of tissue hydration in scenarios where anti-inflammatory effects and matrix stabilization are prioritized over cellular activation.

The distinct kinetic and morphological profiles of LMHMW-HA versus HMW-HA formulations suggest potential for indication-specific selection: formulations promoting sustained cellular proliferation and migration (LMHMW-HA) may be preferable in scenarios requiring tissue regeneration and cellular recruitment, while formulations inducing rapid matrix stabilization and hydration-mediated closure (HMW-HA) may be advantageous when immediate structural support is needed. The complementary nature of these mechanisms also supports rationale for dual molecular weight formulations that sequentially address multiple repair phases, as demonstrated in recent ex vivo studies showing enhanced outcomes with combined molecular weight approaches [26,27]. Future investigations should employ three-dimensional skin models and in vivo wound healing assays to validate these in vitro observations under physiological conditions, including evaluation of dermal-epidermal interactions, angiogenesis, and immune responses. Additionally, comparative studies using matched concentrations would enable direct assessment of relative efficacy. Understanding the molecular weight-dependent effects of HA formulations combined with bioprotective agents such as trehalose may facilitate development of next-generation biostimulatory products optimized for specific clinical indications in tissue repair and aesthetic medicine. Future studies should also investigate whether addition of trehalose to HMW-HA formulations would modulate the hydration-mediated closure mechanism or provide complementary metabolic protection during tissue stabilization phases.

While this study was conducted using an in vitro keratinocyte monolayer model to investigate cellular repair mechanisms, the observed molecular weight-dependent effects of HA formulations may have hypothetical implications for in vivo wound healing processes. The distinct repair patterns—gradual cellular proliferation and migration with LMHMW-HA versus rapid hydration-mediated edge approximation with HMW-HA—could potentially correspond to different functional requirements across wound healing phases, where early inflammatory activation and subsequent matrix stabilization are both necessary for successful tissue restoration. However, it must be emphasized that the present study does not directly demonstrate wound healing efficacy, and extrapolation from this in vitro cellular repair model to complex in vivo wound healing requires caution. Future studies employing three-dimensional tissue models and in vivo wound healing assays would be necessary to validate whether the cellular mechanisms identified here translate to enhanced clinical wound healing outcomes.

## 4. Materials and Methods

### 4.1. Cellular Procedures and Study Methodology

The study was conducted in an in vitro cellular repair model using Normal Human Epidermal Keratinocytes (NHEK), which are primary cells from adult donors. All experiments were performed at Complife Italia S. r. l. (San Martino Siccomario, Italy) under standardized laboratory conditions. The objective was to evaluate the ability of two hyaluronic acid-based formulations to promote cellular repair processes.

Two separate experiments were carried out independently:

(1) Resteem X—a formulation containing low- and medium-high-molecular-weight range hyaluronic acid (200–400 and 1200–1500 kDa) combined with trehalose;
(2) Resteem L—a formulation containing high-molecular-weight range hyaluronic acid (1800–2600 kDa).

Each test was performed in triplicate and included untreated control cultures (CTR−). All quantitative measurements were performed in triplicate (*n* = 3) for each experimental condition, ensuring reproducibility and statistical validity.

Human keratinocytes were maintained in Dulbecco’s Modified Eagle Medium (DMEM) supplemented with 10% fetal bovine serum and incubated at 37 °C in a humidified atmosphere containing 5% CO_2_ until full confluence was reached. After achieving confluence, an artificial linear lesion was mechanically introduced into the monolayer to simulate a controlled cellular injury (cell repair model). The formulations were applied immediately after injury, and cellular responses were monitored over 48 h.

### 4.2. Preparation of Hyaluronic Acid Formulations

The tested formulations were supplied by URGO Sp. z o.o. (Warsaw, Poland) as sterile, ready-to-use medical devices designed for intradermal injection in aesthetic medicine.

Resteem X contained a combination of low- and medium-high-molecular-weight range hyaluronic acid at a total concentration of 25 mg/mL, with a weight ratio of 20:80 between the low-molecular-weight fraction (200–400 kDa) and the medium-high-molecular-weight fraction (1200–1500 kDa). Trehalose was present at a concentration of 1% (*w*/*v*, equivalent to 10 mg/mL). The complete composition included: sodium hyaluronate (1200–1500 kDa and 200–400 kDa), trehalose, sodium chloride, sodium dihydrogen phosphate dihydrate, dibasic sodium phosphate dodecahydrate, and water for injection.

Resteem L contained high-molecular-weight range hyaluronic acid at a total concentration of 20 mg/mL. The complete composition included: sodium hyaluronate (1800–2600 kDa), sodium chloride, sodium dihydrogen phosphate dihydrate, dibasic sodium phosphate dodecahydrate, dodecahydrate, and water for injection.

For in vitro testing, both formulations were diluted in cell-culture medium to obtain final concentrations established as non-cytotoxic in preliminary assays. For Resteem X, concentrations of 0.5%, 0.25% and 0.1% were used, corresponding to final hyaluronic acid concentrations of 125 µg/mL, 62.5 µg/mL, and 25 µg/mL, respectively. For Resteem L, concentrations of 0.25%, 0.1% and 0.05% were tested, corresponding to final hyaluronic acid concentrations of 50 µg/mL, 20 µg/mL, and 10 µg/mL, respectively. The concentration ranges for each formulation were determined empirically through preliminary cytotoxicity screening, starting from 10% and performing serial 1:2 dilutions until non-cytotoxic concentrations were identified. The different concentration ranges between Resteem X and Resteem L reflect the distinct tolerability profiles of low- and medium-high ranges versus high range molecular weight hyaluronic acid in keratinocyte cultures, with higher concentrations of HMW-HA requiring lower dilutions to maintain cell viability. This approach ensured that each formulation was tested at its optimal non-cytotoxic range, allowing for accurate assessment of repair-promoting activity without confounding cytotoxic effects.

### 4.3. Cytotoxicity Assessment (MTT Assay)

A preliminary cytotoxicity evaluation was performed to determine non-toxic concentration ranges suitable for subsequent efficacy testing. The MTT assay (3-[4,5-dimethylthiazol-2-yl]-2,5-diphenyltetrazolium bromide) was used following standard ISO 10993-5 guidelines [29].

Keratinocytes underwent exposure to serial dilutions of each formulation for 24 h. Following incubation, cells underwent washing with Dulbecco’s phosphate-buffered saline (DPBS, pH 7.1–7.7) followed by incubation with 1 mg/mL MTT solution for 3 h at 37 °C, and the produced formazan crystals were solubilized in isopropanol. The absorbance was quantified at 540 nm utilizing a microplate reader, and cell viability was presented as a percentage relative to untreated control groups.

### 4.4. Induction of Controlled Cellular Injury (Cell Repair Model)

Following cytotoxicity testing, confluent keratinocyte monolayers were scratched using a sterile pipette tip to create a defined acellular gap representing a mechanical injury. Immediately after wounding, culture media containing each formulation at the selected concentrations were added. Untreated wells served as negative controls.

Cellular repair dynamics were observed at baseline (T0), 2 h (T2 h), 8 h (T8 h), and 24 h (T24 h) post-injury. Additional monitoring continued up to 48 h to document complete closure of the cellular gap. Each condition was performed in triplicate.

### 4.5. Imaging and Quantitative Analysis

At each observation point, cultures were examined using an optical microscope (Nexcope NIB620, Ningbo, China) equipped with a MIChrome camera (Tucsen, Fuzhou, China). Micrographs were captured at 4× magnification. The inter-edge distances were quantified by measuring the inter-edge distance at three equidistant points along the scratch using ImageJ software (version 1.53, National Institutes of Health, Bethesda, MA, USA). For each replicate, three measurements were taken at distinct locations along the lesion to ensure representative sampling of the entire wound area. Mean values ± standard deviation were determined for every time interval and formulation concentration.

The progression of cellular repair was expressed as the percentage reduction in inter-edge distance relative to baseline (T0). Representative images illustrating the morphological changes during repair were obtained for each experimental condition.

### 4.6. Statistical Analysis

Values are presented as mean ± standard deviation (SD) derived from three separate experiments (*n* = 3). Differences between the treated cultures and untreated controls were evaluated at corresponding time points using the two-tailed Student’s *t*-test. Furthermore, differences between the initial baseline (T0) and later time points within each experimental group were assessed using the paired *t*-test. Significance was established at *p* < 0.05. All statistical analyses were executed utilizing Microsoft Excel (Microsoft Corporation, Redmond, WA, USA) employing the software’s inherent statistical functions.

### 4.7. Ethical Statement

This study did not involve human participants or animal subjects. Normal Human Epidermal Keratinocytes (NHEK) used in this research were commercially obtained primary cells from certified biological material suppliers. According to European Union Directive 2010/63/EU on the protection of animals used for scientific purposes and international guidelines for in vitro research, the use of commercially available de-identified primary cell cultures does not require additional ethical approval. All experimental procedures were conducted exclusively on in vitro cell cultures at Complife Italia S. r. l. (San Martino Siccomario, Italy) in accordance with Good Laboratory Practice (GLP) guidelines. No personal or identifiable human data were collected or processed in this study.

## 5. Conclusions

This study demonstrates that two hyaluronic acid-based formulations with distinct molecular weight profiles—LMHMW-HA combined with trehalose (Resteem X) and HMW-HA alone (Resteem L)—promote keratinocyte repair through distinct mechanisms in an in vitro model. Both the low- and medium-high-molecular-weight range HA formulations (Resteem X; 200–400 and 1200–1500 kDa) and the high-molecular-weight range HA formulation (Resteem L; 1800–2600 kDa) significantly enhanced gap closure compared to untreated controls, with no cytotoxic effects observed at any tested concentration. The LMHMW-HA formulation promoted gradual gap closure over 48 h with increased cellular density, suggesting enhanced proliferation and migration. In contrast, the HMW-HA formulation induced faster initial lesion edge contraction with complete closure at approximately 30 h, accompanied by transient pericellular swelling consistent with hydration-mediated effects. These findings indicate that LMHMW-HA with trehalose and HMW-HA formulations promote keratinocyte repair through distinct kinetic and morphological patterns in an in vitro scratch assay model. The gradual closure with increased cellular density observed with LMHMW-HA and the rapid closure with transient pericellular swelling observed with HMW-HA suggest different modes of action consistent with mechanisms reported in the literature. While these preliminary observations provide foundational characterization of the two formulations, future studies employing molecular and cellular assays (proliferation markers, migration-specific assays, extracellular matrix analysis) will be necessary to definitively establish the underlying mechanisms. The molecular weight-tailored approach may offer therapeutic potential in aesthetic and regenerative medicine applications requiring enhanced tissue repair.

## 6. Patents

Panzieri F. (2021). Injectable composition including hyaluronic acid and use of the said composition. Patent No. US 20210315804A1). U.S. Patent and Trademark Office.

## Figures and Tables

**Table 1:** Mean inter-edge distances (µm ± SD) and percentage change (Δ vs. T0) in control (CTR−) and Resteem X-treated cultures (0.5%, 0.25%, 0.1%). Values are presented as mean ± standard deviation derived from three separate experiments (*n* = 3).

Time	CTR− (µm ± SD)	Δ vs. T0 (%)	Resteem X 0.5% (µm ± SD)	Δ vs. T0 (%)	Resteem X 0.25% (µm ± SD)	Δ vs. T0 (%)	Resteem X 0.1% (µm ± SD)	Δ vs. T0 (%)
T0	691.4 ± 66.7	—	663.1 ± 27.7	—	659.3 ± 25.1	—	672.6 ± 41.2	—
T2H	685.9 ± 56.2	−0.8	646.8 ± 81.5	−2.5	649.4 ± 84.5	−1.5	665.6 ± 56.1	−1.0
T8H	643.4 ± 31.4	−6.9	512.9 ± 80.2	−22.7	527.3 ± 33.9	−20.0	605.0 ± 31.3	−10.0
T24H	556.3 ± 37.2	−19.5	416.4 ± 69.3	−37.2	467.4 ± 83.2	−29.1	510.7 ± 47.2	−24.1

**Table 2:** *p*-values (Student’s *t*-test) comparing Resteem X-treated cultures to baseline (T0) and to control (CTR−). Values correspond exactly to those in the report.

Time	CTR− (*p* vs. T0)	Resteem X 0.5% (*p* vs. T0)	*p* vs. CTR−	Resteem X 0.25% (*p* vs. T0)	*p* vs. CTR−	Resteem X 0.1% (*p* vs. T0)	*p* vs. CTR−
T2H	0.647	0.534	0.253	0.780	0.297	0.780	0.454
T8H	0.073	0.002	0.000	0.010	0.000	0.010	0.019
T24H	0.001	0.000	0.000	0.000	0.010	0.000	0.037

**Table 3:** Mean inter-edge distances (µm ± SD) and percentage change (Δ vs. T0) in control (CTR−) and Resteem L-treated cultures (0.25%, 0.1%, 0.05%). Values are presented as mean ± standard deviation derived from three separate experiments (*n* = 3).

Time	CTR− (µm ± SD)	Δ vs. T0 (%)	Resteem L 0.25% (µm ± SD)	Δ vs. T0 (%)	Resteem L 0.1% (µm ± SD)	Δ vs. T0 (%)	Resteem L 0.05% (µm ± SD)	Δ vs. T0 (%)
T0	691.4 ± 66.7	—	655.1 ± 22.4	—	658.8 ± 27.7	—	662.8 ± 96.5	—
T2H	685.9 ± 56.2	−0.8	649.0 ± 38.9	−0.9	653.9 ± 90.8	−0.7	661.7 ± 51.9	−0.2
T8H	643.4 ± 31.4	−6.9	515.9 ± 44.5	−21.3	555.0 ± 49.6	−15.8	619.7 ± 77.6	−6.5
T24H	556.3 ± 37.2	−19.5	407.8 ± 40.0	−37.8	456.1 ± 35.7	−30.8	525.0 ± 18.0	−20.8

**Table 4:** *p*-values (Student’s *t*-test) for Resteem L-treated cultures vs. baseline (T0) and vs. control (CTR−). Values exactly as reported in the original dataset.

Time	CTR− (*p* vs. T0)	Resteem L 0.25% (*p* vs. T0)	*p* vs. CTR−	Resteem L 0.1% (*p* vs. T0)	*p* vs. CTR−	Resteem L 0.05% (*p* vs. T0)	*p* vs. CTR−
T2H	0.647	0.655	0.125	0.981	0.382	0.981	0.357
T8H	0.073	0.000	0.000	0.458	0.000	0.458	0.407
T24H	0.001	0.000	0.000	0.003	0.000	0.003	0.037

## Data Availability

The original contributions presented in this study are included in the article. Further inquiries can be directed to the corresponding author.

## Abbreviations

The following abbreviations are used in this manuscript:

ECM	extracellular matrix
IL-1	interleukin-1
KGF	keratinocyte growth factor
GM-CSF	granulocyte-macrophage colony-stimulating factor
HA	hyaluronic acid
HMW-HA	High-molecular-weight hyaluronic acid
LMW-HA	Low-molecular-weight hyaluronic acid
LMHMW-HA	Low- and medium-high-molecular-weight range hyaluronic acid
TLR2/4	Toll-like receptors 2 and 4
AGEs	advanced glycation end-products
DMEM	Dulbecco’s Modified Eagle Medium
MTT	3-[4,5-dimethylthiazol-2-yl]-2,5-diphenyltetrazolium bromide
PBS	phosphate-buffered saline

## References

[1] Bártolo I., Reis R.L., Marques A.P., Cerqueira M.T. (2022). Keratinocyte growth factor-based strategies for wound re-epithelialization. Tissue Eng. Part B Rev..

[2] Bornes L., Windoffer R., Leube R.E., Morgner J., van Rheenen J. (2021). Scratch-induced partial skin wounds re-epithelialize by sheets of independently migrating keratinocytes. Life Sci. Alliance.

[3] Kida M., Fatima I., Rozhkova E., Otero-Viñas M., Wu M., Kalin J.H., Cole P.A., Falanga V., Alani R.M., Sharov A.A. (2024). Inhibition of the CoREST repressor complex promotes wound re-epithelialization through the regulation of keratinocyte migration. J. Investig. Dermatol..

[4] Hegde A., Ananthan A.S., Kashyap C., Ghosh S. (2021). Wound healing by keratinocytes: A cytoskeletal perspective. J. Indian Inst. Sci..

[5] Holt J.R., Zeng W.Z., Evans E.L., Woo S.H., Ma S., Abuwarda H., Loud M., Patapoutian A., Pathak M.M. (2021). Spatiotemporal dynamics of PIEZO1 localization controls keratinocyte migration during wound healing. eLife.

[6] Kawano Y., Patrulea V., Sublet E., Borchard G., Iyoda T., Kageyama R., Morita A., Seino S., Yoshida H., Jordan O. (2021). Wound healing promotion by hyaluronic acid: Effect of molecular weight on gene expression and in vivo wound closure. Pharmaceuticals.

[7] Huang J., Heng S., Zhang W., Liu Y., Xia T., Ji C., Zhang L.J. (2022). Dermal extracellular matrix molecules in skin development, homeostasis, wound regeneration and diseases. Semin. Cell Dev. Biol..

[8] Polizzi A., Leanza Y., Belmonte A., Grippaudo C., Leonardi R., Isola G. (2024). Impact of hyaluronic acid and other re-epithelializing agents in periodontal regeneration: A molecular perspective. Int. J. Mol. Sci..

[9] Tolg C., Messam B.J.-A., McCarthy J.B., Nelson A.C., Turley E.A. (2021). Hyaluronan functions in wound repair that are captured to fuel breast cancer progression. Biomolecules.

[10] Rippa A.L., Kalabusheva E.P., Vorotelyak E.A. (2019). Regeneration of dermis: Scarring and cells involved. Cells.

[11] Gomes R.N., Manuel F., Nascimento D.S. (2021). The bright side of fibroblasts: Molecular signature and regenerative cues in major organs. npj Regen. Med..

[12] Anderegg U., Simon J.C., Averbeck M. (2014). More than just a filler—The role of hyaluronan for skin homeostasis. Exp. Dermatol..

[13] Litwiniuk M., Krejner A., Speyrer M.S., Gauto A.R., Grzela T. (2016). Hyaluronic acid in inflammation and tissue regeneration. Wounds.

[14] Monavarian M., Kader S., Moeinzadeh S., Jabbari E. (2019). Regenerative scar-free skin wound healing. Tissue Eng. Part B Rev..

[15] Mast B.A., Frantz F.W., Diegelmann R.F., Krummel T.M., Cohen I.K. (1995). Hyaluronic acid degradation products induce neovascularization and fibroplasia in fetal rabbit wounds. Wound Repair Regen..

[16] Pang X., Li W., Chang L., Gautrot J.E., Wang W., Azevedo H.S. (2021). Hyaluronan (HA) immobilized on surfaces via self-assembled monolayers of HA-binding peptide modulates endothelial cell spreading and migration through focal adhesion. ACS Appl. Mater. Interfaces.

[17] Du Y., Cao M., Liu Y., He Y., Yang C., Wu M., Zhang G., Gao F. (2016). Low-molecular-weight hyaluronan (LMW-HA) accelerates lymph node metastasis of melanoma cells by inducing disruption of lymphatic intercellular adhesion. Oncoimmunology.

[18] Liu J., Wang B.Y., Liu C.H., Yang C., Zhao B.T. (2024). Proteomic analysis reveals the mechanism that low molecular weight hyaluronic acid enhances cell migration in keratinocyte. J. Pharm. Biomed. Anal..

[19] Chmielewski R., Lesiak A. (2024). Mitigating glycation and oxidative stress in aesthetic medicine: Hyaluronic acid and trehalose synergy for anti-AGEs action in skin aging treatment. Clin. Cosmet. Investig. Dermatol..

[20] Benaroudj N., Lee D.H., Goldberg A.L. (2001). Trehalose accumulation during cellular stress protects cells and cellular proteins from damage by oxygen radicals. J. Biol. Chem..

[21] Mizunoe Y., Kobayashi M., Sudo Y., Watanabe S., Yasukawa H., Natori D., Hoshino A., Negishi A., Okita N., Komatsu M. (2018). Trehalose protects against oxidative stress by regulating the Keap1–Nrf2 and autophagy pathways. Redox Biol..

[22] Alvarez-Peral F.J., Zaragoza O., Pedreno Y., Argüelles J.C. (2002). Protective role of trehalose during severe oxidative stress caused by hydrogen peroxide and the adaptive oxidative stress response in *Candida albicans*. Microbiology.

[23] Sarkar S., Davies J.E., Huang Z., Tunnacliffe A., Rubinsztein D.C. (2007). Trehalose, a novel mTOR-independent autophagy enhancer, accelerates the clearance of mutant huntingtin and α-synuclein. J. Biol. Chem..

[24] Zhang X., Chen S., Song L., Tang Y., Shen Y., Jia L., Le W. (2014). mTOR-independent, autophagic enhancer trehalose prolongs motor neuron survival and ameliorates the autophagic flux defect in a mouse model of amyotrophic lateral sclerosis. Autophagy.

[25] Muto J., Fukuda S., Watanabe K., Dai X., Tsuda T., Kiyoi T., Kameda K., Kawakami R., Mori H., Shiraishi K. (2023). Highly concentrated trehalose induces prohealing senescence-like state in fibroblasts via CDKN1A/p21. Commun. Biol..

[26] Chmielewski R., Lebiedowska A., Barańska-Rybak W. (2025). Evaluation of the anti-glycation protective effect of an injectable product based on a combination of two different ranges of molecular weights of hyaluronic acid and trehalose on human skin explants. Int. J. Mol. Sci..

[27] Chmielewski R., Lebiedowska A., Barańska-Rybak W. (2025). Assessment of the curative anti-glycation properties of a novel injectable formulation combining dual-weight hyaluronic acid (low-and mid/high-molecular weight) with trehalose on human skin ex vivo. Int. J. Mol. Sci..

[28] Gao Y., Sun Y., Yang H., Qiu P., Cong Z., Zou Y., Song L., Guo J., Anastassiades T.P. (2019). A low molecular weight hyaluronic acid derivative accelerates excisional wound healing by modulating pro-inflammation, promoting epithelialization and neovascularization, and remodeling collagen. Int. J. Mol. Sci..

[29] (2009). Biological Evaluation of Medical Devices—Part 5: Tests for In Vitro Cytotoxicity.

